# Psychosocial recommendations for the care of children and adults with epidermolysis bullosa and their family: evidence based guidelines

**DOI:** 10.1186/s13023-019-1086-5

**Published:** 2019-06-11

**Authors:** K. Martin, S. Geuens, J. K. Asche, R. Bodan, F. Browne, A. Downe, N. García García, G. Jaega, B. Kennedy, P. J. Mauritz, F. Pérez, K. Soon, V. Zmazek, K. M. Mayre-Chilton

**Affiliations:** 1 University Hospitals Birmingham NHS Trust, Solihull Hospital, Solihull, B91 2JL, UK; 2 Universital Hospitals Leuven, Leuven, Belgium; 3 DEBRA Norge and person living with JEB, Stavanger, Norway; 4 California State University, Fullerton, CA USA; 5 Our Lady’s Children’s Hospital Crumlin, Dublin, Ireland; 6 Guy’s and St Thomas’ Hospitals NHS Foundation Trust, London, UK; 7 DEBRA Spain, Marbella, Spain; 8 Psychology graduate and person living with EBS, Liverpool, UK; 9 The University Medical Center Groningen, Groningen, the Netherlands; 10 DEBRA Chile, Santiago, Chile; 11 Great Ormond Street Hospital NHS Foundation Trust, London, UK; 12 DEBRA Croatia, Zagreb, Croatia; 13 DEBRA International, Vienna, Austria

**Keywords:** Epidermolysis bullosa, Psychosocial, Guideline, Recommendations, Psychological, Social, Family, Professionals

## Abstract

Epidermolysis Bullosa (EB) is a group of rare genetic disorders resulting in skin fragility and other symptoms. Commissioned by DEBRA International and funded by DEBRA Norway, this evidence-bases guideline provides recommendations to optimise psychosocial wellbeing in EB.

An international multidisciplinary panel of social and health care professionals (HCP) and people living with EB was formed. A systematic international literature review was conducted by the panel following the Scottish Intercollegiate Guidelines Network (SIGN) methodology. The resulting papers underwent systematic selection and critique processes. Included papers were allocated to 6 different outcome groups to allow data synthesis and exploration: quality of life, coping, family, wellbeing, access to HCP and pain. Based on the evidence in those papers, recommendations were made for individuals living with EB, family and caregivers and HCP working in the field.

Few studies have investigated interventions and which factors lead to better outcomes, but general recommendations can be made. EB is a complex disease impacting enormously on every aspect of psychosocial life. People and families living with EB need access to multidisciplinary support, including psychological guidance, in order to improve quality of life and psychosocial wellbeing. Interventions should stimulate social participation to prevent isolation. People with EB and their families should be able to access a supportive network. HCP should be well supported and educated about the complexity of EB. They should work collaboratively with those around the individual with EB (e.g. schools, employers etc.) to provide psychosocial opportunity and care.

Attention should be paid to the psychosocial impact of EB as well as physical needs. Directions for research are indicated.

**Electronic supplementary material:**

The online version of this article (10.1186/s13023-019-1086-5) contains supplementary material, which is available to authorized users.

## Introduction

Epidermolysis Bullosa (EB) is a group of rare genetic disorders, the primary manifestation is the formation of blisters and erosions in response to mechanical trauma [1]. While there are currently over 30 known subtypes of EB, there are four primary types including EB Simplex (EBS), Dystrophic EB (DEB), Junctional EB (JEB), and Kindler syndrome (KS) [1, 2]. EB can be the result of either inherited or spontaneous dominant mutations, as seen in most forms of EBS and Dominant DEB (DDEB); or from inherited recessive mutations as in rare forms of EBS, Recessive DEB (RDEB), JEB, and KS [2].

The severity and scope of EB varies widely by type and subtype. The mildest forms of EBS and DDEB may be limited to localised bullae (blisters) and wounds which can be very painful, impacting quality of life (QoL) [1]. More severe forms of EBS, RDEB, and JEB can result in generalised bullae and erosions, with significant extracutaneous involvement, which may require the care of a large multidisciplinary team. Potential complications of more severe EB include, but are not limited to, sepsis, anaemia, failure to thrive, pseudosyndactyly, contractures, and squamous cell carcinoma [3–12]. Average life expectancy for those with severe RDEB is about 30 years of age [13]. With the exception of infant mortality, seen in rare cases of EBS, and more commonly in JEB, life expectancy is thought to be unaffected in other forms of EB [4, 6].

Currently there is no cure for EB; all known treatments at this point are experimental. The mainstay of EB management continues to be supportive in nature, with symptom control and wound care at the centre. Wound care can be a very painful and lengthy process, lasting up to several hours a session, done daily or every other day for most patients [14]. As a result of the time it takes to care for the medical needs and the pain and discomfort that is inherent with the condition, EB can significantly impact all domains of a patient and family’s life. This includes interactions with family, friends, and peers, education, employment and leisure time [15–17]. Due to the profound impact of EB, it is understandable that many individuals living with EB may struggle with physiological suffering and psychosocial sequelae.

Psychosocial health is a broad term defined by the American Psychological Association as ‘describing the intersection and interaction of social, cultural and environmental influences on the mind and behaviour’ [18]. Whilst some individuals will be able to successfully navigate their lives without psychosocial intervention or assistance from the health care team, many would likely benefit from assessment and intervention. Unfortunately, at this time, there is little in the way of guidance for clinicians regarding how to best care for the psychosocial health of patients and families affected by EB. When we consider the extent to which their lives can be affected, and the impact that psychosocial health can have on one’s physical health, this is a pressing need for those in the EB community.

DEBRA International (DI) therefore sponsored the development of psychosocial clinical practice guidelines (CPGs) for individuals and families living with EB. In 2016 an expert panel was convened by DI comprising clinicians from several countries with expertise related to social care and mental health and EB, people living with EB and family members from the international EB community.

An extensive systematic literature search and synthesis was conducted by the expert panel. Based on the synthesis of the literature and priorities raised by the EB community, six outcomes were found to be relevant with regards to the psychosocial health of those affected by EB: QoL, coping, family, well-being, access to health care professionals (HCPs) and pain. Whilst the concepts of QoL, coping and well-being are complex with many interpretations and some overlap, the expert panel agreed on the following published definitions for each of these three concepts:

- ❖ ***QoL:*** defined as an ‘individual’s perception of their position in life, in the context of the culture and value systems in which they live and in relation to their goals, expectations, standards and concerns. It is a broad ranging concept affected in a complex way by the person’s physical health, psychological state, level of independence, social relationships, personal beliefs and their relationship to salient features of their environment’ [19].
- ❖ ***Coping:*** defined as ‘thoughts and behaviours that people use to respond to internal or external stressful demands’ [20].
- ❖ ***Well-being:*** defined as the ‘balance point between an individual’s resource pool and the challenges faced’ [21].

An evidence-based discussion of the impact of EB on QoL, coping, family, well-being, access to HCPs and pain follows. The synthesis and discussion include suggestions for potential prevention, intervention strategies and suggestions for future research.

### Objectives of the CPG

- ❖ Provide guidance on the psychosocial needs of people with EB, their families and those who care for them.
- ❖ Outline the current state of the science on the psychosocial implications of EB on patients and their family members.
- ❖ Include recommendations for care.
- ❖ Identify gaps in knowledge to encourage future research.

***Users*** These guidelines are intended for professionals working to help those living with EB, employers, teachers, stakeholders and policy makers. They are for those who care for people with EB (all types) and their families.

### Target groups

- ❖ Professionals caring for EB patients and their families.
- ❖ EB patients of all ages and diagnosed with any of the four major types of EB: EB Simplex, Junctional EB, Dystrophic EB and Kindler Syndrome.
- ❖ Families of people with EB.

## Methods

The expert panel of multidisciplinary HCPs and people living with EB was co-ordinated through Debra International (DI) through a voluntary membership. The international panel represented clinical, social or personal experience of EB covering both adult and paediatric knowledge (Additional file 1. shows panel membership). The panel voted for their clinical representative (indicated here as the first two authors, chair and co-chair). During the development of the CPG two people living with EB and four HPCs had to resign from their roles due to changes in commitments or conflict of interest (CoI). Two new HCPs were accepted on to the panel. Panel members were encouraged to be involved in all stages of the CPG development (Additional file 1).

**Fig. 1:**
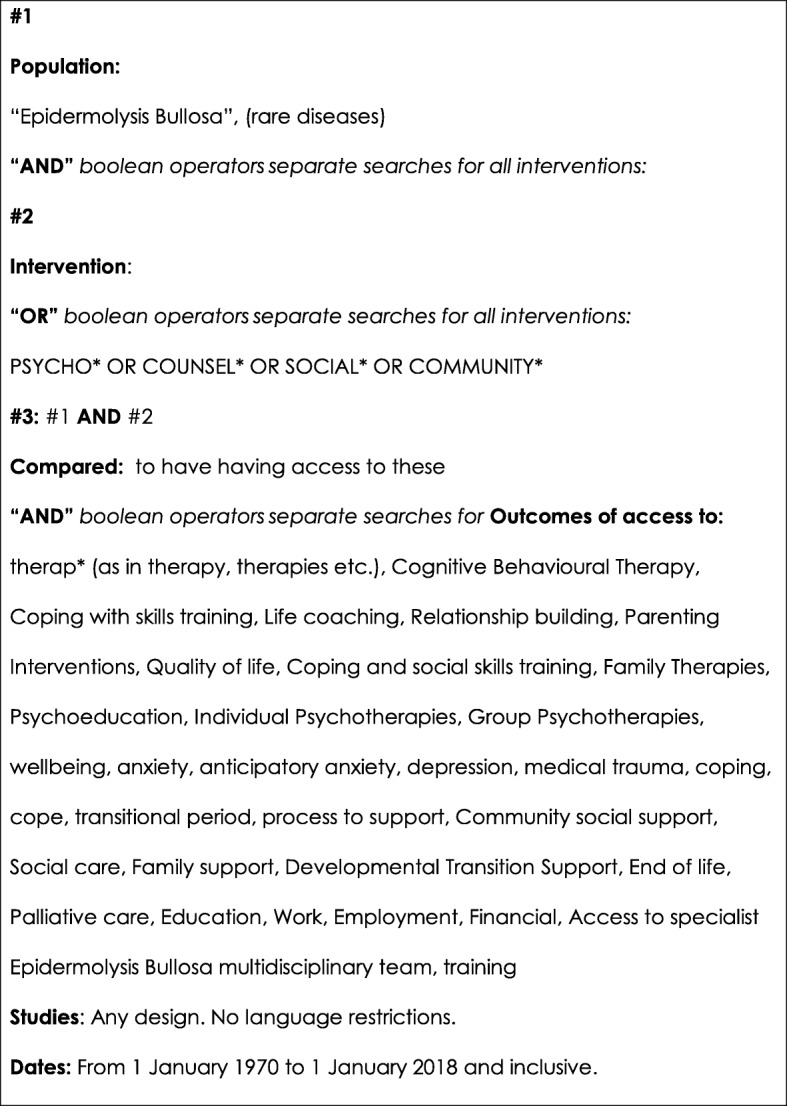
Search terms and inclusion criteria

Following the Scottish Intercollegiate Guidelines Network [22] (SIGN) methodology, the panel decided on the research question “Can psychosocial support help people to cope with EB?” The concepts of QoL, cope and well-being were defined to help the panel and analyses of the literature. The focus of the project was decided by considering the relevant “PICOs” (Fig. 1): **P**articipants were the target populations, **I**nterventions was to have psychosocial support or psychotherapy or social worker support, **C**ompared to not having this support available, **O**utcomes and study design. These terms were informed by: priorities raised by people living with EB in a focus group conducted at the Croatia DI Congress 2016; a preliminary literature search conducted by a PhD study and presentation from an expert in the field [23]; and further discussion about the meaning of “psychosocial” and “cope” amongst the panel.

### Literature search process

Preliminary guideline database searches were undertaken in 2016: https://www.ahrq.gov/gam/updates/index.html; www.g-i-n.net; www.clinicaltrials.gov and SIGN [22]. Eight guidelines in other rare conditions were identified. There were no EB guidelines covering psychosocial care.

**Fig. 2:**
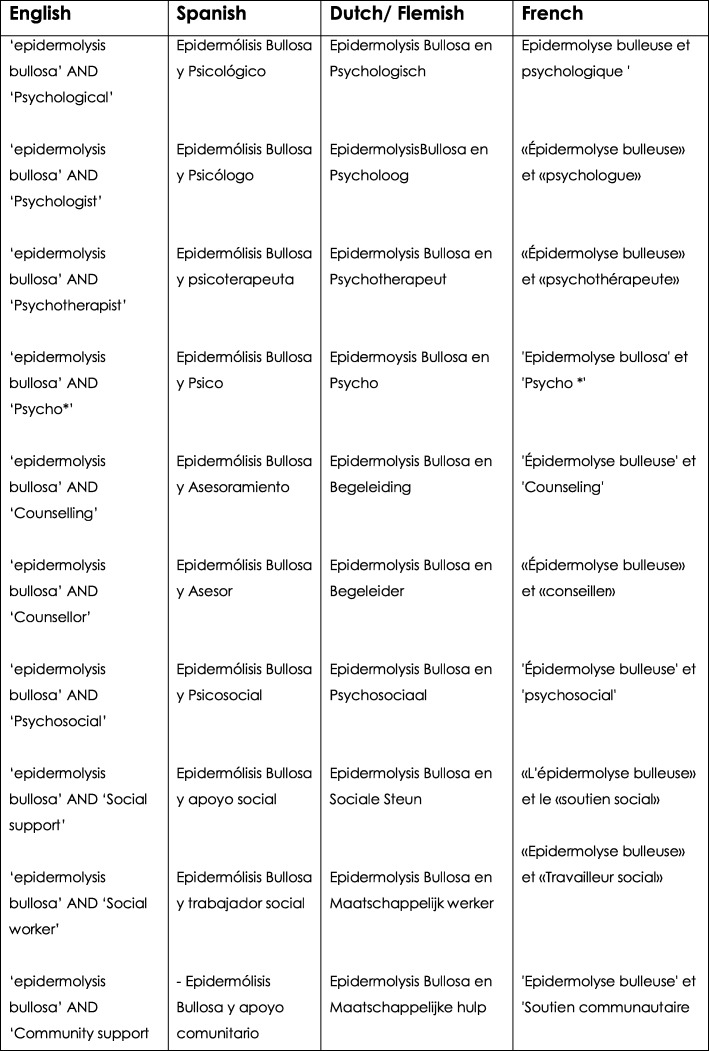
Translated Key search terms

A systematic literature search regime was conducted by librarians and nine of the panel members. Twelve electronic search engines were accessed: Medline (PubMed MeSH), Embase Emtree PsychInfo, CINHAL, Scopus, Dialnet, Google academic, British Nursing Index, Health management Information consortium (HMIC), Allied and complementary medicine (AMED), Health business elite and the main search engine for the National Institute for Health and Care Excellence (NICE). Figure 1 shows the search terms, inclusion criteria utilising PICOS, and the boolean AND and OR operators used to combine these terms as appropriate. Terms were kept broad, translated and searched for in all engines (Fig. 2).

**Fig. 3:**
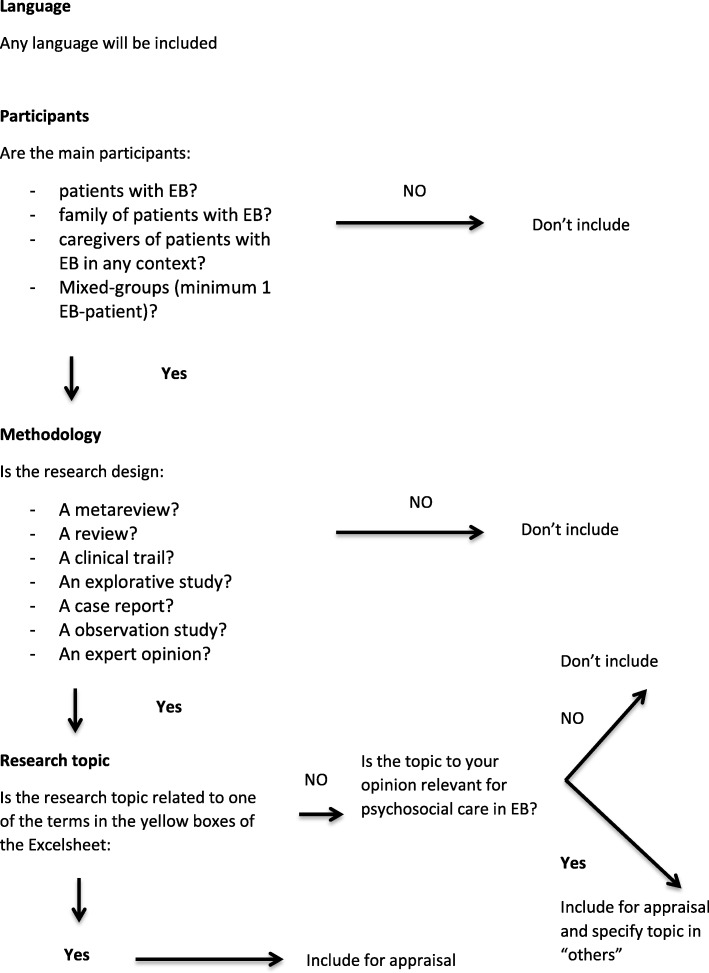
Flow-chart to filter articles based on title and abstract

Cited reference searches were conducted on eligible papers. Ongoing search re-runs were continued up to 1st July 2018. Every member of the panel conducted the filtration step and research appraisal. All articles meeting the criteria for appraisal were included (Additional file 2 copy of filtration and appraisal tool). The flowchart illustrates the inclusion filters applied to all articles identified by the searches (Fig. 3). These filters were discerned from each paper’s abstract and title and, in cases of uncertainty, through KM and SG examining the full articles. One paper in German was filtered by a German speaking volunteer through DI. Articles not meeting the criteria were excluded.

Book chapters, abstracts, presentations and papers which were unpublished or did not meet the methodological filters were retained as gray literature. These were examined to provide context or consider divergence within the main recommendations. Four additional articles were recommended through the review process, these were screened and 3 were excluded as they did not meet the criteria of inclusion.

#### Research appraisal

All published papers meeting the filters were then subject to a systematic quality appraisal and risk of bias assessment. The appraisal tool (Additional file 2 copy of filtration and appraisal tool) was modified from the Critical Appraisal Skills Programme [24] tools, GRADE [25] tools and a quality assessment framework [26]. This allowed both quantitative and qualitative research to be appraised using one list of questions, yielding one quality rating scale to allow a comparison of studies as required. The precision and statistical consistency could not be evaluated as the EB articles had no statistical values. In most cases there was more than 50% risk of bias as it is a rare condition; there are no double blind randomised clinical studies and most participants would know they have EB. Study limitations were taken into account through the appraisal tool.

Each paper was appraised by two panel members to ensure consistency and a research quality score was obtained; the higher the value the better the quality of the paper. To prevent biases and promote reliability, precautions were taken; in cases where a panel member had authored a paper this was reviewed by other members of the panel. The level of bias was also measured in percentage values following GRADE [25] (Additional file 2) and all papers were graded in accordance to SIGN [22] method “Level of evidence and Grades of Recommendations” 1++ to 4 and Grade A to D. In cases of disagreement a third appraiser KM or SG was assigned, and uncertainty resolved.

The papers were then divided into outcome topics. These were identified through the panel ranking the outcomes’ relative importance anonymously using an online survey (doodle.com), based on the Grading of Recommendations Assessment Development and Evaluation [25] (GRADE). These outcomes provided the focus of the guideline: QoL, well-being, cope, pain (management), family (support or counselling) and HCPs (access to service or support.

Some papers related to more than one outcome, so were included in all that were relevant. Two or more panel members focused on an outcome and summarised all relevant papers. They presented the emerging evidence and discussed the recommendations elicited at a meeting held in Salzburg at EB-CLINET 2017. This summary addressed: what were the recommendations emerging from the appraised research? What was the quality of the research? Were there any gaps in this research? Did the gray literature on this outcome support the emerging recommendations or suggest something different? What was the level of agreement of the members summarising the papers on this outcome? Outcome summary tables were presented to highlight the population subtypes, numbers of subjects, type of study, percentage quality and risk of bias and in accordance to SIGN [22] method “Level of evidence and Grades of Recommendations” 1++ to 4 of the papers for each outcome.

The panel rated the strength of each recommendation, the percentage bias, desires and undesired effects, costs related to benefits and its feasibility to implement. The GRADE [25] framework tool for recommendations was completed to standardise the wording used to formulate recommendations for each outcome. Summaries were circulated to the panel and final feedback was included.

Best practice points were identified and intended to assist guideline users by providing short pieces of advice which are seen as essential to good clinical practice [25]. The Appraisal of Guidelines for Research & Evaluation (AGREE) II tool [27] was consulted to increase the quality of practice guidelines in rare diseases and this CPG acknowledges existing guidelines by signposting with the symbol ^⇒^ through this manuscript.

Twenty-three expert independent referees were invited to review the CPG, 11 signed a roles and responsibility agreement and CoI forms prior to the anonymised manuscript being circulated. The inclusion of experts from the EB community reflected the multi-disciplinary and international nature of the guideline (Additional file 1 shows author and reviewer panels). Reviewers were asked to comment primarily on the comprehensiveness and accuracy of the interpretation of the evidence base supporting the recommendations, and on how this fitted with their experiences. They were given 6 weeks to do this. This process of review is in accordance with SIGN [22]. The chair and co-chair discussed the reviewers’ feedback with the guideline panel: except for the two authors who had originally declared a CoI. Each point was addressed and changes made; if no change was made, the reason for this was recorded. The panel conducted a final proof read of the manuscript before submission.

## Results

**Fig. 4:**
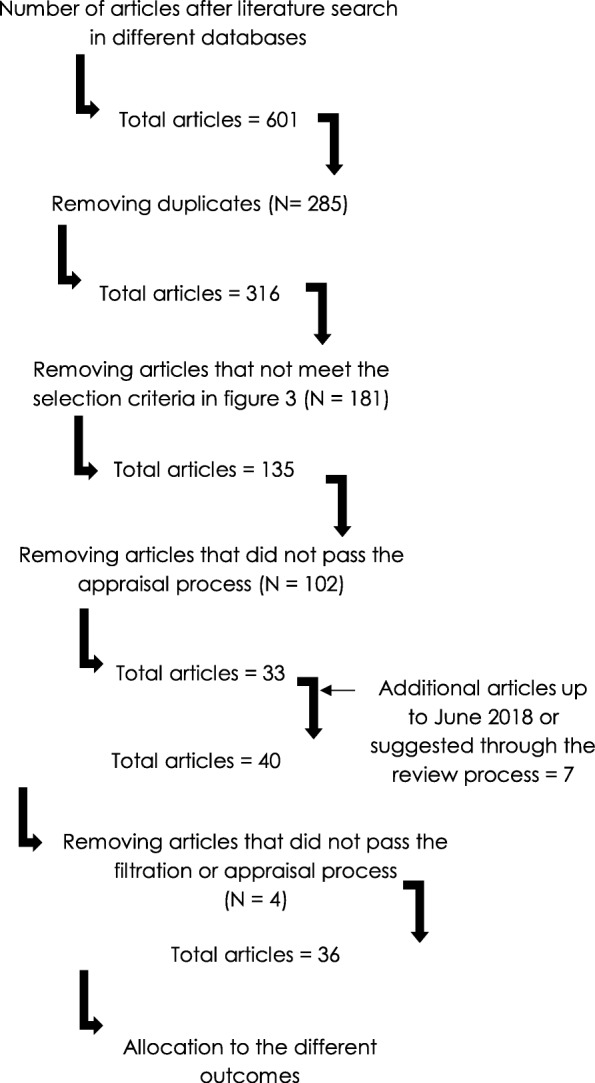
Flow of information through the evidence evaluation process

A total of 34 articles were eligible for inclusion in the final review. Figure 4 shows the flow of data through the stages of the search, filtering and appraisal process.

**Fig. 5:**
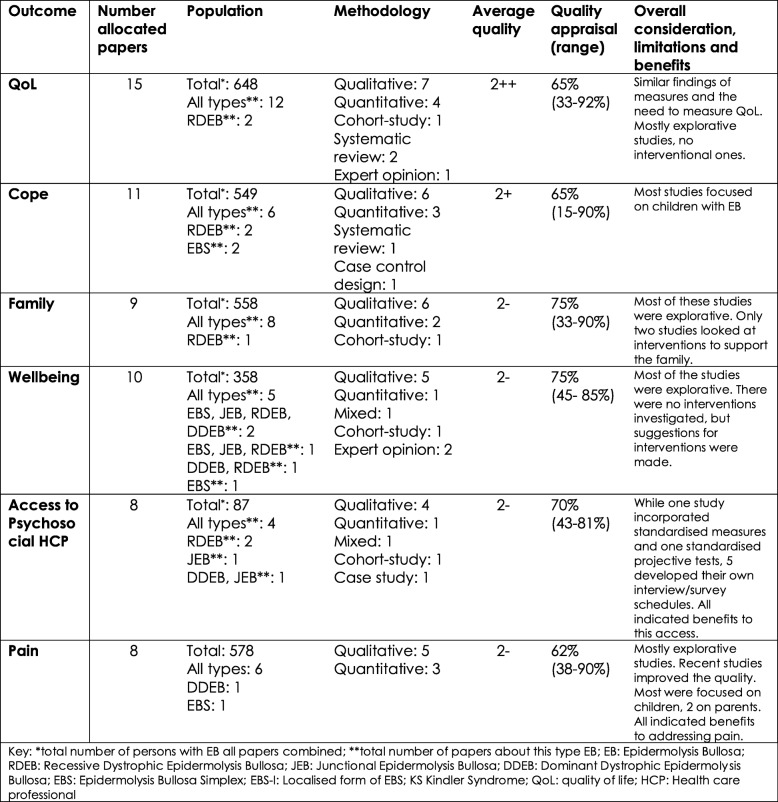
Overview evidence per outcome

These papers were then allocated to the six different outcomes: Fig. 5 gives an overview of the selected articles after the appraisal process and the allocation to each outcome. QoL was the outcome with the most allocated papers (*n* = 15) and Access to HCPs and Pain had the least (*n* = 8). Furthermore, Fig. 5 shows the overall number of participants, and the number of people with different types of EB, in the papers allocated to each outcome. It describes their methodologies and the average quality of the papers per outcome. Finally, it demonstrates the average score (and range) on the appraisal criteria and a brief general consideration, limitations and benefits per outcome.

## Recommendations

The recommendations are outlined and discussed within the context of professionals providing A. psychosocial care for the individual with EB, B. psychosocial care for the family and caregiver, then C. self-care for professionals working with those affected by EB (Tables 1, 2 and 3).

**Table:** 

Recommendation Summary	Grade strength	Quality of evidence *(Average)*	Key references
A. Psychosocial care for individuals living with EB			
We strongly recommend easy access to psychosocial support to improve QoL	B✓	2++	[28–32]
We strongly recommend psychosocial support to improve well-being	C	2+	[32, 33–36]
We strongly recommend gaining access to psychosocial support for the whole family	C✓	2-	[37–39]^α^
We recommend psychosocial support to help with pain	C	2-	[3, 7, 17, 30, 37, 40–44]
We strongly recommend psychosocial support to help coping with EB	C✓	2-	[34, 37, 45, 46]
We strongly recommend psychosocial support from a multidisciplinary Health Care Team	C✓	2-	[17, 29, 38, 40, 46–51]
B. Psychosocial care for family and care givers of people with EB			
We strongly recommend access to psychosocial family support to improve the family QoL	B	2+	[29, 30, 38]
We strongly recommend psychosocial support to improve family well-being	C	2-	[30, 36, 50, 52–54]
We strongly recommend family counselling in order to prevent family breakdown	C✓	2-	[16, 17, 30, 38, 50, 52, 55]
We strongly recommend psychosocial support to help the whole family to cope with living with EB	C	2-	[16, 17, 30, 51]
We recommend psychosocial support to reduce emotional burden during daily painful procedures	C	2-	[35, 37, 45, 46]
We strongly recommend easy access to a multidisciplinary expert team for the whole family	C	2-	[17, 29, 39, 50, 51]
C. Self-care for professionals working with those affected by EB			
We strongly recommend psychosocial expertise to help people to cope with living with EB	C	2-	[17, 34, 46]
We strongly recommend a collaborative patient-professional relationship	C	2-	[40, 49, 56]
We strongly recommend offering support for professionals working in EB	C	2-	[40, 49, 56]
KEY: EB: Epidermolysis Bullosa; QoL: quality of life; n: number of participants; ^α^ gray literature			
Grades	Descriptions in accordance to SIGN [22]		
B	A body of evidence including studies rated as 2++, directly applicable to the target population, and demonstrating overall consistency of results; or Extrapolated evidence from studies rated as 1++ or 1+		
C	A body of evidence including studies rated as 2+, directly applicable to the target population and demonstrating overall consistency of results; or Extrapolated evidence from studies rated as 2++		
Ratings	Descriptions in accordance to SIGN [22]		
2++	High quality case control or cohort studies with a very low risk of confounding or bias and a high probability that the relationship is causal		
2+	Well conducted case control or cohort studies with a low risk of confounding or bias and a moderate probability that the relationship is causal		
2-	Case control or cohort studies with a high risk of confounding or bias and a significant risk		
✔ Recommended best practice based on the clinical experience of the guideline development group [22]

**Table 1:** Recommendations summary for the Psychosocial care of individuals living with EB

Recommendations	Population	Grade strength	Quality of evidence *(Average)*	Quality of evidence	Key references
i. We strongly recommend easy access to psychosocial support to improve Quality of life (QoL)					
A multidisciplinary approach in treating EB improves QoL for individuals with EB • Psychological support and close monitoring of EB improves QoL. • They facilitate participation in social activities. • Patients with all types of EB including EBS report a great impairment in QoL due to restrictions in physical and social activities.	✓ Adults, children (*n* = 12/185) EBS; JEB; DDEB; RDEB; KS Unclear if adults, children or both (*n* = 43/134) EBS; JEB; DDEB; RDEB; Adults, children (*n* = 120/248) EBS; JEB; DDEB; RDEB Review of inherited & autoimmune blistering diseases	B	2++	1- 2+ 2+ 2- 1-	[29] [30] [31] [28] [32]
ii. We strongly recommend psychosocial support to improve well-being					
To promote self-efficacy and support around body image to aid psychological well-being • Having access to knowledge and resources about EB can help people have a greater role in managing their EB. This self-management can help improve well-being. • Improved self-efficacy and locus of control, as well as support around body-image could help to develop a more positive sense of well-being. For support during transition periods in life (school transitions, transition into adulthood) • Communication and education about EB to improve people’s understanding. • Support from families, EB healthcare professionals and DEBRA.	Review of inherited &autoimmune blistering diseases Adults (*n* = 87) RDEB, DEB, EBS Children 10–14 years old (*n* = 11) EBS (autosomal recessive) Young male adults (aged 21–35 years) with RDEB (*n* = 5) and EBS (*n* = 2) Observational report	C	2+	1- 2+ 2+ 4 4	[32] [33] [34] [35] [36]
iii. We strongly recommend gaining access to psychosocial support for the whole family					
People diagnosed with EB should be referred for psychosocial support as early as possible in childhood or in adulthood, if the person with EB wishes • To support the family unit. Encourage supportive network for the family, for example: • Education about EB for others • Provide access to DEBRA (or other EB support groups)	✓ Children (*n* = 11/82) EBS; JEB; DDEB; RDEB Children (*n* = 16) JEB Adult (*n* = 1) RDEB	C	2-	2- 2- 4	[37] [38] [39]^α^
iv. We recommend psychosocial support to help with pain					
Pain is present for most children and adults with EB (all types) with profound psychosocial impact: • Activity related pain can significantly affect psychosocial well-being and QoL (e.g. fear of/actual pain restricting social activities, affecting relationships with family and friends). • Treatment related pain can make managing EB harder and link to procedural anxiety. Adequate holistic pain management is essential as a focus for helping people with EB: • Following pain guidelines. • Offering approaches to help people with EB cope emotionally. • Help with managing the impact of pain and the interlinked cycle of pain and psychosocial challenges.	Adults, children(*n* = 374) EBS, JEB, DDEB, RDEB Adults (*n* = 6) JEB, DDEB Children; (*n* = 11) EBS, JEB, DDEB, RDEB, Adults (*n* = 30) children (*n* = 27) EBS Children/families (*n* = 70) type of EB unclear Adults (*n* = 43) EBS, JEB, DDED, RDEB Unclear if adult/child (*n* = 40) EBS, JEB, DDEB, RDEB Best practice guideline Children (*n* = 11) EBS, JEB, DDEB, RDEB Adult (*n* = 1) RDEB	C	2-	2+ 2- 2- 2- 2- 2+ 2+ ⇒ 2+ 3	[3] [40] [37] [42] [41] [30] [44] [7]^α^ [17] [43]
v. We strongly recommend psychosocial support to help cope with living with EB					
People with EB need support to cope with EB, and their ways of coping need to be supported by others: participation in social life needs to be supported • Such as at school, the community, friendships, employment. • Aid access to supportive networks. • Public education campaigns to help those around them to understand EB and their needs. Promote a sense of self-management of their EB • This can help bring a sense of control over certain aspects of the disease/treatment and pain. Build social skills and communication • Help in learning how to communicate about EB to others and within the family unit.	✓ Children (*n* = 27) DDEB; (*n* = 28) RDEB Children 10–14 years old (*n* = 11) EBS (autosomal recessive) Children (*n* = 11/82) EBS; JEB; DDEB; RDEB Children (*n* = 24) EBS; JEB; DDEB; RDEB	C	2-	2+ 2+ 2- 2-	[45] [34] [37] [46]
vi. We strongly recommend psychosocial support from a multidisciplinary Health Care Team					
Encourage access to, and a collaborative ‘working together’ relationship with, an expert multi-disciplinary team of professionals. • Facilitate access to multidisciplinary professional support for medical and psychosocial care across the lifespan. • At both specialist centres and community services	✓ Adults, children (*n* = 15) RDEB Children (*n* = 21) EBS; JEB; DDEB; RDEB Children (*n* = 11/82) EBS; JEB; DDEB; RDEB HCPs (*n* = 33) 30 stakeholders (HCPs, and 9 with EB RDEB, DDEB, EBS) Adults (*n* = 6) JEB, DDEB Children and Adults (*n* = 20) EBS, JEB, DEB Children (*n* = 16) JEB Children (*n* = 20) EBS, JEB, RDEB	C	2-	1- 2+ 2+ 2+ 2- 2- 2- 2- 2- 2+	[29] [48] [51] [17] [46] [49] [40] [38] [48] [47]
Key: EB: Epidermolysis Bullosa; RDEB: Recessive Dystrophic Epidermolysis Bullosa; JEB: Junctional Epidermolysis Bullosa; DDEB: Dominant Dystrophic Epidermolysis Bullosa; EBS: Epidermolysis Bullosa Simplex EBS-I: Localised form of EBS; KS Kindler Syndrome; QoL: quality of life; n: number of; ^α:^ gray literature; ⇒this is an EB guideline					
Grades	Descriptions in accordance to SIGN [22]				
B	A body of evidence including studies rated as 2++, directly applicable to the target population, and demonstrating overall consistency of results; or Extrapolated evidence from studies rated as 1++ or 1+				
C	A body of evidence including studies rated as 2+, directly applicable to the target population and demonstrating overall consistency of results; or Extrapolated evidence from studies rated as 2++				
Ratings Descriptions in accordance to SIGN [22]					
1-	Meta-analyses, systematic reviews, or RCTs with a high risk of bias				
2++	High quality case control or cohort studies with a very low risk of confounding or bias and a high probability that the relationship is causal				
2+	Well conducted case control or cohort studies with a low risk of confounding or bias and a moderate probability that the relationship is causal				
2-	Case control or cohort studies with a high risk of confounding or bias and a significant risk				
3	Non-analytic studies, e.g. case reports, case series				
4	Expert opinion				
✔ Recommended best practice based on the clinical experience of the guideline development group [22]

**Table 2:** Recommendations summary for the Psychosocial care of Family and caregivers of people living with EB

Recommendations	Population	Grade strength	Quality of evidence *(Average)*	Quality of evidence	Key references
i. We strongly recommend access to psychosocial family support to improve the family QoL					
Early psychosocial support to improve *QoL* of the *family* unit for all subtypes EB and children with high infantile mortality: • As caregivers QoL may also be impacted. • Psychological support and close monitoring helps. • Support is essential for family of palliative patients with EB.	Adults, children; 11 studies EBS; JEB; DDEB; RDEB Adults, children (*n* = 125/185) EBS; JEB; DDEB; RDEB; KS Children (*n* = 16) JEB	B	2+	1- 2+ 2-	[29] [30] [38]
ii. We strongly recommend psychosocial support to improve the family well-being					
Support for the *family* to reduce emotional burden of caring for someone with EB and improve *well-being* for the *family* unit*:* • Home nursing can provide much needed relief and support for primary caregivers and could reduce the need for hospital admission. • Actively assist in seeking counselling before the family unit is irreparably destroyed. • Provide information about the nature, course and outcome of EB. • Provide training in the management of patient symptoms. • Access to Social media and face to face EB support groups might be beneficial for families. Promoting family well-being can help the family enhance their strong and positive influence for those living with EB • The way the family reacts to EB can be psychologically assimilated by the person with EB, particularly children. • Acceptance of the EB by the family is important and can make it more bearable for the patient.	Adults, children (*n* = 15) RDEB Children, Adults (*n* = 374/ 425) EBS; JEB; DDEB; RDEB Adults, children (*n* = 125/185) EBS; JEB; DDEB; RDEB; KS Adults, children (*n* = 25, 14 children, 11 adults) RDEB, EBS Adult – personal experience	C	2-	2+ 2+ 2- 4 2+ 4	[50] [52] [30] [36] [53] [54]
iii. We strongly recommend family counselling in order to prevent family breakdown					
To help prevent the *family* unit *breakdown,* for the *family* of all EB subtypes: • Strengthen *family* relationships. To prevent *family* emotional breakdown or distress • Support in managing life with EB. To prevent *parents’* emotional breakdown or distress • Specially provide support for single parents with a child living with EB.	Children, Adults (*n* = 374/ 425) EBS; JEB; DDEB; RDEB Adults, children (*n* = 15) RDEB Children (*n* = 11/82) EBS; JEB; DDEB; RDEB	C	2-	2+ 2+ 2-	[52] [50] [17]
	Adults, children (*n* = 28/42) EBS; JEB; DDEB; RDEB Children (*n* = 16) JEB Children, Young Adults; (*n* = 63/138) EBS; DDEB; RDEB+			2- 2- 2-	[16] [38] [55]
	Adults, children (*n* = 125/185) EBS; JEB; DDEB; RDEB; KS			2-	[30]
iv. We strongly recommend psychosocial support to help the whole family to cope with living with EB					
Specialist home based psychosocial support for the *family* of all EB subtypes can help promote strategies to *cope*: • Help access counselling to promote the intra-*family* communication. • Access help to manage EB and economic burden. • Promote good relationships between the family • Provide a home care program for respite, or support handing over physical care to others.	Children (*n* = 21) EBS; JEB; DDEB; RDEB Adults, children (*n* = 125/185) EBS; JEB; DDEB; RDEB; KS Adults, children (*n* = 28/42) EBS; JEB; DDEB; RDEB Children (*n* = 11/82) EBS; JEB; DDEB; RDEB	C	2-	2+ 2- 2- 2-	[51] [30] [16] [17]
v. We recommend psychosocial support to reduce emotional burden during daily painful procedures					
Psychosocial support needs for parents and family to reduce the emotional burden of caring for someone living with EB who has severe *pain:* • Offer psychological support for caregivers. Parents/care givers can struggle with ‘causing pain’ due to dressing changes and wanting to protect their child from pain. This is very difficult emotionally for parents. • Pain can negatively affect relationships within the family and with friends. Help optimise pain management techniques. • Parents/carers can find it difficult to see people in severe pain. Aid access to respite, independent carers and promote independence with dressings.	✓ Children (*n* = 11/82) EBS; JEB; DDEB; RDEB Adults, children; (*n* = 57) EBS-I Adult (*n* = 6/20) JEB, DDEB	C	2-	2+ 2- 2-	[17] [42] [40]
vi. We strongly recommend easy access to a multidisciplinary expert team for the whole family					
Provide access to recognised expert support and training for the whole family • Provide appropriate treatment and training or refer to national EB experts. • Referring to with the DEBRA or EB support network may help.	Adults, children; 11 studies EBS; JEB; DDEB; RDEB Adults, children (*n* = 15) RDEB Children (*n* = 21) EBS; JEB; DDEB; RDEB Children (*n* = 11/82) EBS; JEB; DDEB; RDEB Adult (*n* = 1) RDEB	C	2-	1- 2+ 2+ 2+ 4	[29] [50] [51] [17] [39]^α^
Key: EB: Epidermolysis Bullosa; RDEB: Recessive Dystrophic Epidermolysis Bullosa; JEB: Junctional Epidermolysis Bullosa; DDEB: Dominant Dystrophic Epidermolysis Bullosa; EBS: Epidermolysis Bullosa Simplex EBS-I: Localised form of EBS; KS Kindler Syndrome; QoL: quality of life; n: number of; ^α:^ gray literature; ⇒this is an EB guideline					
Grades	Descriptions in accordance to SIGN [22]				
B	A body of evidence including studies rated as 2++, directly applicable to the target population, and demonstrating overall consistency of results; or Extrapolated evidence from studies rated as 1++ or 1+				
C	A body of evidence including studies rated as 2+, directly applicable to the target population and demonstrating overall consistency of results; or Extrapolated evidence from studies rated as 2++				
Ratings Descriptions in accordance to SIGN [22]					
1-	Meta-analyses, systematic reviews, or RCTs with a high risk of bias				
2+	Well conducted case control or cohort studies with a low risk of confounding or bias and a moderate probability that the relationship is causal				
2-	Case control or cohort studies with a high risk of confounding or bias and a significant risk				
4-	Expert opinion				
✔ Recommended best practice based on the clinical experience of the guideline development group [22]

**Table 3:** Psychosocial management recommendations for professional working with EB

Recommendations	Population	Grade strength	Quality of evidence *(Average)*	Quality of evidence	Key references
i. We strongly recommend psychosocial expertise to help people to cope with living with EB					
Access to EB specialised care • Nurturing a good relationship between professionals, family and person with EB • Training for non-EB professionals	Children 10–14 years old (*n* = 11) EBS (autosomal recessive) Children (*n* = 11/82) EBS; JEB; DDEB; RDEB Children (*n* = 24) EBS; JEB; DDEB; RDEB	C	2-	2+ 2- 2-	[34] [17] [46]
ii. We strongly recommend a collaborative patient-professional relationship					
• Training for professionals in working collaboratively with patients.	HCPs (*N* = 33)	C	2-	2-	[56]
iii. We strongly recommend offering support for professionals working in EB					
• To promote well-being for the healthcare professional • Emotional support is necessary: Personal support but also on an organisational level. • Importance of Professionals self-care: awareness, support to do this and access to clinical supervision. • Important to not work in isolation: the need to link in with an MDT and to feel equipped through information and education to help with psychosocial needs.	Key Stakeholders (*N* = 30) Key Stakeholders (*N* = 30) Adults (*N* = 6) JEB, DDEB	C	2-	2- 2- 2-	[56] [49] [40]
Key: EB: Epidermolysis Bullosa; RDEB: Recessive Dystrophic Epidermolysis Bullosa; JEB: Junctional Epidermolysis Bullosa; DDEB: Dominant Dystrophic Epidermolysis Bullosa; EBS: Epidermolysis Bullosa Simplex EBS-I: Localised form of EBS; KS Kindler Syndrome; HCPs: Health care professionals; MDT: multidisciplinary team; n: number of; ^α:^ gray literature; ⇒this is an EB guideline					
Grades	Descriptions in accordance to SIGN [22]				
C	A body of evidence including studies rated as 2+, directly applicable to the target population and demonstrating overall consistency of results; or Extrapolated evidence from studies rated as 2++				
Ratings	Descriptions in accordance to SIGN [22]				
2-	Case control or cohort studies with a high risk of confounding or bias and a significant risk				

## A. Discussion of psychosocial care for individuals living with EB

### Quality of life (*Grade: B)* ✓

Two observational studies investigated the impact of EB on QoL in adults and children with all types of EB [28, 30]. They affirm that EB can have a severe impact on QoL with the physical symptoms of EB (such as itching, pain and stinging) having the highest impact on QoL for adults, along with restrictions to social activities and embarrassment due to their skin [28, 30]. Children identified embarrassment as the highest impacting factor, followed by physical symptoms and restrictions to play and activities. People with EBS particularly identified how pain restricted social activities [42].

Further work needs to be established regarding facilitating people with EB to participate in social activities, to enable greater QoL and well-being Although no interventions are investigated in the research, recommendations are that the provision of psychological support and the measurement and monitoring of QoL could be beneficial for people with EB [30, 32, 57] (Table 1i.).

### Wellbeing (*Grade: C*)

People with EB can have a negative body image due to the visibility of the condition and recognition of being different from others [33–34]. A negative body image can be related to poorer psychological well-being. Women and younger people with EB may struggle more than men in this respect [33]. For some the visibility of EB, such as on hands and face, can invite scrutiny from others [34, 37, 46] as can the invisibility of EB for others [37]. The social impact of EB due to visible difference or exclusion from activities was most strongly identified in children’s accounts of living with EB [34].

Although there were no intervention-based studies within the literature, the authors recommend that interventions are needed to enable people with EB to have a stronger belief in being able to gain control over aspects of their condition. This can be associated with well-being and improved body image, particularly in children with EB [33]. Having access to knowledge and resources about EB and inviting people with EB to take a collaborative rather than ‘being done to’ approach to their care, can help them to have a greater role in managing their EB. Specific support to help with body image is also advocated.

Transition periods of life were identified as potentially stressful for people with EB, impacting on their well-being and coping. Examples of transitions include changing classes at school and having to inform others about EB [34]. Although there is a lack of research into adults’ experiences, expert opinion and experienced clinicians identify that transitions in adulthood can also give rise to additional stress, such as transitioning from school to university/college or the work place and when developing new relationships. Children with EB and their families can have anxieties around transitioning from paediatric to adult healthcare settings, and regarding building relationships and trust within a new healthcare environment [58]. Transition should be seen as a process, not a single event, and partnership working between the clinical staff in both health settings, community services and family are essential in facilitating a smooth transition [58]. The provision of education, information and support for the individual and their family, from EB professionals, is recommended to help improve psychological well-being during these transition periods [34, 36, 58] (Table 1.ii.).

### Family (*Grade: C*) ✓

There is limited research on the interaction between the impact of EB on the family unit and the person living with EB, and none looking at this into adulthood. The impact of EB on the family unit can, however, be profound and recommendations regarding the family’s psychosocial needs are covered below. Based on clinical experience and the expertise in the panel, it is likely that the nature of EB will impact on early attachment relationships and bonding within the family unit. The need for psychosocial support for the person with EB as part of a family unit is therefore advocated for all types of EB diagnosis [37] (Table 1.iii.). People with EB can struggle with a sense of difference compared to others in their family, and their family life may be different to that of other families. Empowering those around the individual to understand EB may help. Having access to peer relationships with other families and people living with EB may help provide a sense of being understood, alongside education being available to those around the person with EB and their family; though not all individuals or families may wish to access this resource [38, 39]. DEBRA events and online social media support networks could also be beneficial [35, 36].

### Pain (grade: C)

The strongest conclusion to be drawn from this review is that pain is a key theme for many people affected by EB. The limited literature indicates that pain can have a negative effect on children and adult’s QoL and psychosocial well-being [59]. Most people with EB have pain due to their skin wounds and other extracutaneous symptoms. This can be severe and unrelenting, with aspects of treatment often being linked to painful procedures. The pain itself can have profound effects, being associated with frustration, embarrassment, anxiety, sadness and, especially for children, fear [3, 14, 37, 40, 42]. Pain impacts on friendships and family relationships. Activity related pain can restrict many areas associated with positive psychosocial well-being and coping; such as social participation, having access to peer relationships, occupation, meaningful activities, and maintaining friendships [3, 37, 40]. ^⇒^ It is therefore important that support is given to manage not just the pain itself, but also its impact psychologically and socially, and that all possible is done to manage pain in accordance with pain management guidelines [7].

There is much within general non-EB specific literature on psychosocial techniques for managing pain. There are reviews regarding interventions for paediatric pain [60, 61], other sources [62, 63] and the ^⇒^British Pain Society Guidelines for pain management programmes for adults which may be helpful resources [64].

These psychosocial aspects to pain management could be applicable to the EB population but no intervention studies explore this generalizability. Indeed, people with EB describe coping with pain by “blocking it out” or distraction [40, 42] and some studies suggest a limited use of techniques such as deep breathing and relaxation by people with EB [40]. We recommend that pain management is a priority within clinical management and research aimed at improving the psychosocial well-being of people with EB (Table 1.iv.).

### Coping (*Grade: C*) ✓

People with EB develop individualised ways of coping with the condition. Themes within the literature associated with coping include having a sense of self-management, therefore control, over aspects of their condition and treatment; particularly for children with EB [37]. This is echoed in the recommendation for a collaborative approach to healthcare outlined below.

Wherever possible support and help to participate as fully as possible in social life needs to be advocated. This includes at school, within the community and, as an adult, within opportunities for employment and a role within society. Whilst there is an ongoing struggle and need to achieve a balance between activity and risk of skin damage for many people with EB, access to social participation is highlighted by both adults and children with EB as important parts of their coping with the condition [32, 34, 45]. Such activity also allows access to other forms of coping such as friendships and support networks. In childhood this socialisation and mastery of activities is important in meeting developmental milestones and psychosocial development.

One of the barriers to participation in activities and social roles can be a perceived or actual lack of understanding of others regarding the needs of people with EB. Support and education in this regard can help, such as public campaigns and, more specifically, support within schools and for employers to understand how they can assist [34]. Communication within the family unit, alongside confidence in communicating about EB to others is important, particularly in childhood for building social confidence [37, 45, 46] (Table 1.v.).

### Access to professional healthcare (*Grade: C*) ✓

Multiple studies suggest that being able to access a MDT of professionals, providing treatment and advice on the medical and psychosocial aspects of EB across the lifespan, is key for improving psychosocial well-being, coping and QoL for people with EB [38, 40, 47–50]. Having a team of people who can work collaboratively with the person with EB and, especially in the case of children with EB, their family and carers, can help provide a sense of support and ease the burden of EB in everyday life [29, 37, 50, 51]. This is also echoed in the other recommendations around education and advice to improving self-management of EB to aid a sense of self-efficacy and control over the condition. Having professionals who are trained in recognising, understanding and helping with psychosocial issues is paramount and may also help in forming a supportive collaborative relationship [47, 49, 56] (Table 1.vi.).

## B. Discussion of psychosocial care for family and care givers of people living with EB

### Quality of life (grade: B) ✓

Life with EB has a significant impact on the entire family and can lead to an impairment in the QoL for both the child and parents [17, 29, 46, 52, 53, 56]. The main determinants associated with QoL for the family of a child with EB are [1] the severity, extent, unpredictable course and (in) visibility of the disease [2], the pain and poor QoL of the child [3], restrictions in employment and leisure time [4], never being off duty and [5] ignorance and lack of skills of care providers [17, 29, 30, 52, 51]. Findings show early psychosocial assessment and monitoring can improve QoL of the entire family of children with EB, regardless of which sub-type and the expected life span [17, 29, 30, 38] (Table 2 .i.).

### Well-being (grade: C)

Many studies have identified the impact of EB on the physical and emotional well-being of parents and caregivers. This includes difficulties in organizing care, problems within their own relationship, having less energy, the unpredictability of the disease and associated difficulties with planning, both in the short and long-term. These problems can affect the whole family, impacting on each member’s ability to achieve their desires and significantly lowering their level of life satisfaction [17, 51, 65] (Table 2. ii.).

Several studies emphasise the importance of early, extensive and long-term support for parents or caregivers which could involve [1]: home nursing to provide relief and help for primary caregivers and could reduce the need for hospital admission [2], provision of information on the nature, course and outcome of the patient’s disease [3], training of relatives in the management of patient symptoms and in the reinforcement of relatives’ social networks [4], and the use of social media [30, 36, 50, 52]. (Table 2. ii.). A study further explores how online communities could be a particularly helpful way for people with EB to share experiences and gain social support in a manageable way across the world [64].

The reaction of family members to the condition seems to be psychologically assimilated by their children and can impact on them as adults living with EB. This shows that it is important to make parents aware of how their reaction to their child’s illness affects the well-being of the whole family [53, 54] (Table 2. ii.).

### Breakdown of the family unit (grade: C)

The potential impact of EB on the QoL and well-being of parents with children who have EB, increases the risk of a breakdown of the entire family unit. To prevent a family breakdown, several studies recommended helping families managing life with EB, strengthening family relationships and especially supporting single parents or female carers who may require more support [16, 17, 30, 38, 50, 52]. It is particularly recommended that parents place importance on, and pay attention to, securing their own leisure time, holidays and social life to prevent a breakdown of the family unit [55] (Table 2. iii.).

### Access to healthcare providers and EB networks (C) ✓

Access to health professionals and EB networks is recommended, where patients and caregivers can receive appropriate treatment, information and training regarding the day-to-day management of their EB, and have the possibility to discuss personal experiences; for example at family weekends or workshops for parents and patients [17, 29, 39, 50, 51, 66, 67] (Table 2. vi.).

### Coping (grade: C)

Parents experience great stress due to inadvertently inflicting pain on their child when managing their daily care. The challenge of ‘switching’ roles between being caregiver and parent can require increased efforts to cope, not only with physical tasks but also cognitive and emotional challenges [17, 51, 65, 68]. Support programs or a care manager should [1] encourage parents to establish a normal routine for the child and family [2] dedicate time for themselves and as a couple to help coping [3] create a support network with the spouse, extended family, medical staff and respite opportunities from care [16, 17, 69] (Table 2. iv.).

### Pain (*Grade: C*)✓

*Coping* with *pain* is interlinked with living with EB from birth. *Pain* can result in negative effects on relationships within the family [40, 42]. It is the primary concern for caregivers who helplessly observe the wounds on the child’s body and worry about causing pain as a consequence of providing the care their child requires [17, 40, 65]. Often parents separate their emotions during painful tasks to cope [17]. Alternatively, they do not get involved in the daily dressing routines, which can have a positive impact on the family unit [40]. Formal expert assistances to support people living with EB to become more independent with dressings, as well as opportunity to access to outside carers, would support the family unit [40] (Table 2. v.).

## C. Discussion of self-care for professionals working with those affected by EB

### It is important that people with EB have access to health care professionals with appropriate expertise and experience in EB (grade: C)

Access to health care professionals within the hospital and community has been recommended, though most of the studies in this area focus on supporting children with EB [49] (Table 3. i). Professionals need to feel they have adequate expertise and training to manage the complexities of EB. There will naturally be limits to the resources available and it is important to identify when other services may be better placed to offer support. Increased awareness and education is needed to equip non-EB specialist community services to respond to the wide range of physical and psychosocial needs of individuals with EB and their caregivers. Online community platforms could form a helpful way to disseminate EB related factual information [64], though reliance on online and telehealth platforms may need to be avoided when physical medical examination by specialists forms a crucial part of EB medical care.

### Health care professionals need to aim for collaborative patient-professional relationships

Studies have also made recommendations regarding the process of supporting adults with EB [14, 38, 40, 70]. Physicians need to acknowledge “the active role of the patients as an informed, involved and interactive partner in the treatment process” [70]. Professionals providing medical intervention need to support patients to develop confidence and motivation to use their own skills and knowledge, enabling them to take effective control over managing their condition. Adults with EB often view themselves as “experts yet also valuing the expertise of others” [40]. EB care needs to be viewed as “a partnership” between individuals with EB and healthcare professionals. Striking a balance between maintaining and valuing self-efficacy in managing EB, whilst promoting access to support and specialist intervention, is very important. Professionals providing these services would benefit from training in working with people with rare conditions. In particular, the process of supporting assertive and well informed patients, as well as patients who decline or struggle to engage with healthcare services (Table 3. ii).

### Healthcare professionals working with people with EB should work in experienced teams providing support for themselves (*Grade: C*)

Clinicians working in the area also benefit from access to professional support [32]. EB professionals often work in isolation and this is not good for their own psychological health. Access to clinical supervision, skills training in recognising psychosocial difficulties and access to multidisciplinary team support are important considerations. This is an important recommendation to consider the deep emotional impact that HPCs can encounter when working in EB care [71]. A range of professional are involved in EB care at specialist hospital, community and voluntary settings; it is important that all involved have access to shared support and supervision. Issues around professional limitations, difficulties and boundaries need to be acknowledged and discussed openly rather than managed in isolation (Table 3. iii).

## Conclusions

These guidelines provide evidence-based recommendations to optimise the care for people living with EB and their families. EB is a disease that impacts on many different aspects of life. Besides the extensive medical aspects, psychosocial well-being is also highly affected. Many factors influence psychosocial well-being and create a complex interaction between disease-specific features, individual characteristics, environmental issues and socio-economic circumstances.

Starting from an evidence-driven approach, these guidelines form a framework with recommendations that can be used by people living with EB, their families and caregivers and professionals. After a comprehensive systematic literature search, the main conclusion we can make is that evidence from interventional studies is limited. Most existing research is explorative in focus, aiming to investigate the impact of EB, rather than interventions which help. Therefore, these guidelines provide general recommendations that can be used as a basis to start an individual-focused approach for supporting people with EB and their families, and to inform future research.

There is a good understanding of which parts of life are difficult for people with EB. The management of pain is a key part of psychosocial well-being, impacting on QoL and coping with EB [40]. Physical discomfort (pain, itching, wound care etc.) plays an important role in the limitations people with EB and their family are experiencing. To cope with this discomfort is a daily mission, demanding a great amount of effort and energy. Whilst there is a ⇒ CPG specifically on pain in EB [7] this CPG utilised only research specifically on pain and psychosocial aspects for people with EB; the Pain specific CPG included non-EB specific papers. There is a lack of intervention studies exploring the application and efficacy of psychosocial techniques for managing pain in EB. Techniques such as pacing and planning activities, relaxation, distraction, managing anxiety, tension and cognitive attributions may all be helpful but there is no clear evidence allowing this formal recommendation.

It seems that living with EB is more bearable when people with EB feel supported and integrated in society. Participation in social life, peer support and a sense of self-realisation are key constructs needed for people with EB to optimise their social well-being and to cope with the difficult effects of EB. In our expert opinion and synthesis of the existing, though limited, evidence base, multidisciplinary professionals, and those supporting and caring for people with EB, should use their expertise to help people living with EB and their families to realise these constructs. People with EB and their caregivers will become the experts in their own situation. A strong collaboration between the scientific driven expertise of professionals and the experience driven expertise of people with EB and their surroundings is recommended.

Starting from infancy, children with EB need to be encouraged to follow their psychosocial developmental milestones, alongside meeting their physical needs. More concretely, this could mean that in early childhood a supportive team should focus on pain management so there can be a secure attachment between children, their parents and the surrounding world. When they are older, they should be able to discover the world, being supported to have a certain grade of mobility and independence: psychologically, socially and where possible physically. As children grow into adulthood, there are a lot of obstacles disturbing their process of psychosocial development and management of EB. Examples of those possible obstacles are: gaining independency, pain as a strong conditioner to developing anticipatory anxiety and general anxiety, absence from school and peer relationships, physical limitations influencing the ability to participate in social activities, difficulties with poor body image, challenging transition periods and identity development. Young adults with EB could wrestle with difficult themes such as sexuality, intimacy, relationships and the sense of reaching their potential in life (self-realisation). Also, for adults with EB, it is not easy to find their way and place in society, but having a sense of belonging, a role, routine and meaningful activity all help with their psychosocial well-being.

The interaction of physical limitations and discomfort caused by EB, with these aforementioned psychosocial developmental tasks that every child and adult have to go through, is complex. There is a scarcity of research about which interventions in childhood give the best outcomes in adulthood, not only in EB, but also in other chronic and invasive diseases. There is also limited research into how adults living with EB and their families can overcome and manage the psychosocial challenges of EB. Most of the recommendations made in this paper are therefore not concrete and a golden standard of care could not be created.

A supportive network seems invaluable. The biggest recommendation we can make to optimise psychosocial well-being of those with EB and their families, is to facilitate participation in society and peer support. Every person has to find his role in life and society and the search for this can be really difficult for those living with EB. These challenges could also extend to family members caring for someone with EB where parents may have to adapt/change family roles to meet the care needs of a child with EB. This can have social and economic implications for the whole family and affect each member’s ability to achieve their own desires.

Parents can experience immense psychological stress due to inflicting pain on their child while managing their daily care. The challenge of being both the parent who wants to protect their child and the caregiver who has to, at times, impose pain due to the child’s physical and medical needs can take a toll on parent’s well-being. Therefore, it is very important to provide early, extensive and long-term support for family members to enable them to cope with the emotional burden that they carry as care providers and regarding their option to bring in outside caregivers or respite. Psychosocial support is recommended to assist families to manage life with EB. Supporting families can help to strengthen family relationships, prevent family breakdown and improve family members QoL and well-being. The literature suggests a link between family members’ reaction to EB, which is assimilated by the child, impacting later on into adulthood. Therefore, if family members are supported, this can also have psychological benefits for the individual living with EB.

There can be a lack of knowledge and understanding about EB in schools, working places, leisure time groups or in peer relationships. Moreover, many social activities or public places are not sufficiently adapted for the needs of people with EB; like supermarkets, shops, restaurants, hotels, transportation. A focus on raising awareness of EB in general and on the psychosocial needs of people with EB is essential. Providing information and training about EB for family members is also recommended to enable them to have a deeper personal understanding about the condition and disease process. Providing family members with information also can equip them to feel more confident in managing situations where EB is not understood. The role of the HCP is important in assisting family members and carers in their understanding of EB and providing them with social support.

Health care professionals can play a supportive role in all of the aforementioned aspects of life and development. They should use their expertise to provide an individualised approach for every person with EB. This can be a demanding task and there are limits to resources available; being aware of the limits and boundaries to their professional responsibilities and capabilities is important. The complexity of this disease is also felt by health care professionals. Feelings of powerlessness and not being able to help enough can be overwhelming, especially given the potentially long-term relationship between professionals and persons living with EB and their family. Therefore, it is essential that health care professionals adopt self-care awareness. They have to take care of themselves in order to be able to care for others. Clinical supervision, having space to reflect and process their experiences of caring and being able to rely on colleagues in a MDT appears necessary. Otherwise the burden of EB can also be hard to carry for health care professionals.

### Limitations of the guideline

The authors acknowledge limitations with this CPG. The panel is formed from international volunteers from the EB community. They are experts in EB but not on CPG methodology. The contribution of a methodology expert to the panel has, however, sought to ensure a high quality robust methodology process. As EB is a rare condition there is a limited numbers of volunteering experts, also people living with EB can be very unwell very quickly, and professional’s clinical responsibilities take priority. This can all add some limitations and challenges but despite this all panel members were encouraged to participate in all stages of developing these recommendations; to promote ecological validity and ensure an inclusive approach.

The CPG focuses only on summarising the published papers and gray literature identified by the search process. This is presented within the context of the primary six outcomes and utilises only EB specific papers. There may well be an overlap regarding the psychosocial difficulties experienced by people with EB and their families, and other conditions or groups of people: such as, for example, people with other physical differences, rare diseases or life-long, painful or and fluctuating conditions. A next step may be to look at the literature in these areas to see if it can be generalised to EB populations. The EB community, in terms of people with EB, their families and professionals, is a small one. The individuals forming the review panel all chose to not be anonymous with their feedback. Whilst the authors’ names were hidden from the review panel, it is likely that some may have known the people writing this review. The implication for the peer review process is unknown. This may be taken as a strength, allowing the writing panel to follow up any comments and points made directly, but it may be a limitation in that the review panel may not be seen as wholly ‘independent’ from the authors. With funding for clinical care being limited, with a keen focus on funding for research into physical care, the authors acknowledge that the review panel may carry their own biases regarding their interpretation of the guidelines, their support for its publication, or may be competitors with biases towards the centres involved in this guideline.

### Future research

This guideline highlights the need for further high-quality research, to understand how best to support and manage the psychosocial needs and coping of people living with EB and their families. Present research goes some way towards understanding the nature of the psychosocial challenges presented by EB, but little is known about how to actually address or manage these effectively. Whilst there is evidence supporting psychosocial interventions for a range of difficulties commonly experienced by people with EB, in areas such as pain management, anxiety and social interactions, there is limited research investigating the application of these to an EB population. There is a lack of focus on adulthood as opposed to managing EB in childhood, and EBS is also under-represented. Two areas of research paucity therefore include studies evidencing interventional approaches and on the management of psychosocial difficulties into adulthood. The rarity of EB may lead to difficulties with small and heterogeneous samples, making quantitative studies complex: international collaborations may be explored as an option to help.

**Fig. 6:**
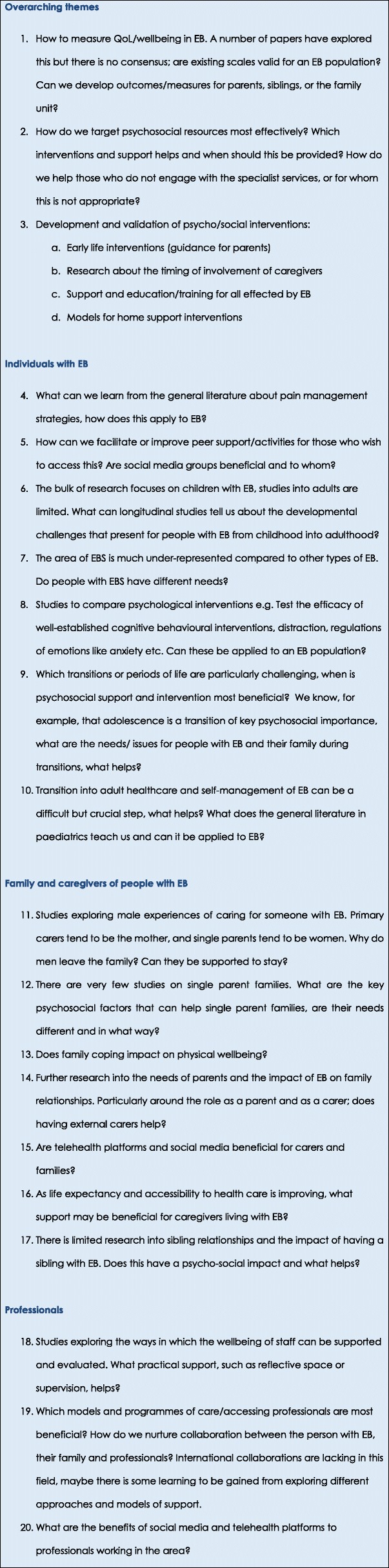
Areas of research for the individual, family, caregivers and professionals

The research questions and areas have emerged as requiring further investigation (Fig. 6):

### Implementation of guideline recommendations and update

DEBRA International aims to ensure that the EB guidelines address the needs of patients internationally. These guidelines will be translated into other languages and patient versions will be made to aid accessibility.

These guidelines could be disseminated and promoted through education for professionals and considering how they could be incorporated into clinical practice. The guidelines were presented at the International DEBRA Congress 2018. The implementation and impact of these recommendations could be monitored and evaluated through audits, education programme registration, and the CPG Evaluation Form: Pre implementation (Additional file 3). The panel recommends sites to pre-audit practice, implement the CPG and re-audit to test improvement, audits tools can be used from SIGN. DEBRA International would value your feedback on the findings to continue to improve CPG quality.

The guidelines should be updated every 3–5 years or if there is a significant breakthrough in EB psychosocial care from the publication date. We recommend a re-run of search terms to see if a full review is warranted at this point.

## Additional files

Additional file 1: Author and Review Panel Membership. (PDF 422 kb)

Additional file 2: Copy of Appraisal and bias Tool. (PDF 638 kb)

Additional file 3 CPG Evaluation Form: Pre implementation. (PDF 138 kb)

## Abbreviations

AGREE: Appraisal of Guidelines for Research & Evaluation
AMED: Allied & complementary medicine
APA: American Psychological Association
CASP: Critical Appraisal Skills Programme
CEO: Chief Executive Officer
CoI: Conflict of interest
CPG: Clinical practice guideline
DDEB: Dominant Dystrophic Epidermolysis Bullosa
DEB: Dystrophic Epidermolysis Bullosa
DEBRA: Dystrophic Epidermolysis Bullosa Research Association
DI: DEBRA International
EB: Epidermolysis Bullosa
EB-CLINET: Epidermolysis Bullosa Clinical Network
EBS: Epidermolysis Bullosa Simplex
EBS-I: Localised form of Epidermolysis Bullosa Simplex
GRADE: Grading of Recommendations Assessment, Development and Evaluation
HCPs: Health Care Professionals
HMIC: Health Management Information Consortium
JEB: Junctional Epidermolysis Bullosa
KS: Kindlers Syndrome
MDT: Multi-disciplinary Team
NICE: National Institute for Health and Care Excellence
PhD: Doctor of Philosophy, an academic grade
PICOS: Participants, Interventions, Comparisons, Outcomes and Study Design
Prof: Professor
QoL: Quality of Life
RDEB: Recessive Dystrophic Epidermolysis Bullosa
SIGN: Scottish Intercollegiate Guidelines Network
