# Supplementary material for: Psychosocial recommendations for the care of children and adults with epidermolysis bullosa and their family: evidence based guidelines
Source: Orphanet J Rare Dis. 2019 Jun 11;14:133. doi: 10.1186/s13023-019-1086-5 (PMC6560722; doi:10.1186/s13023-019-1086-5)
Supplement: Supplementary file 1 — Author and Review Panel Membership. (PDF 422 kb) [file 13023_2019_1086_MOESM1_ESM.pdf]

Psychosocial CPG: Appendix 1

CPG working Panel															
Panel member	Country of origin	Speciality with EB	Role in panel	Focus group	First meeting	Searches	Paper filtration and Appraisal	Outcome summaries	Recommendation meeting	Recommendation Summary	Writing	Panel Review	Incorporation of feedback from panel	Incorporation of feedback from reviewers	Submitting the CPG
K Martin	UK	Senior Clinical Psychologist, adults and families	Chair		✓	✓	✓	✓	✓	✓	✓	✓	✓	✓	✓
S Geuens	Belgium	Clinical Psychologist, adults and paediatrics	Co-Chair		✓	✓	✓	✓	✓	✓	✓	✓	✓	✓	✓
J K Asche	Norway	Person living with EB	PPI Member	✓	✓	✓	✓	✓	✓			✓		× CoI	
R Bodan	USA	Person living with EB	PPI Member			✓	✓	✓	✓		✓	✓	✓		
F Browne	Ireland	Dermatologist	Member			✓	✓	✓	✓			✓			
A Downe	UK	EB CNS	Member			✓	✓	✓	✓		✓	✓	✓	✓	
N Garcia Garcia	Spain	Comminuty Psychologist	Member	✓	✓	✓	✓	✓	✓			✓			
G Jaega	UK	Person living with EB	PPI Member		✓	✓	✓	✓	✓						
B Kennedy	Ireland	Senior clinical psychologist, paediatrics	Member		✓	✓	✓	✓	✓		✓	✓	✓	✓	
P J Mauritz	Netherlands	Child Development Therapist	Member	✓	✓	✓	✓	✓	✓		✓	✓	✓		
F Perez	Chile	Comminuty Psychologist	Member				✓	✓	✓		✓	✓			
K Soon	UK	Clinical Psychologist, paediatrics	Member		✓	✓	✓	✓	✓						
V Zmazek	Croatia	Person living with EB	PPI Member		✓	✓	✓	✓	✓						
K Mayre-Chilton	UK	DI CPG coordinator, Specialist adult research dietitian	Project manager/ Member	✓	✓	✓	✓	✓	✓	✓	✓	✓	✓	× CoI	✓

Key: UK- United Kingdom; USA- United States of America; EB- Epidermolysis bullosa; CNS- EB Clinical Nurse Specialist; DI- DEBRA International; PPI- Patient and Public involved (People living with EB); CoI- Conflict of interest; ✓- involved in this development step; ×- not involved in this

Reviewer Panel List						
K Moss	UK	Psychoanalytic psychotherapist with Adults	GSTT	CoI		
S Butterworth	UK	Regional Community Support	DEBRA UK	CoI	✓	*
G Gößnitzer	Austria	Psychologist	DEBRA Austria			*
J Finnigan	New Zealan	EB CNS	DEBRA New Zealand			
S Cassidy	New Zealan	EB CNS	DEBRA New Zealand			
K Begum	UK	EB CNS	HoE			
A King	USA	Occupational Therapist lead	Phoenix Childrens			
M Zmazek	Croatia	Person living with EB	DEBRA Croatia			

Key: UK- United Kingdom; EB- Epidermolysis bullosa; CNS- EB Clinical Nurse Specialist; GSTT- Guy's and St Thomas' Hospitals NHS Fooundation Trust; BCH- Birmingham Children's Hospital; HoE- Heart of England Foundation Trust, Solihull Hospital; CoI- Conflict of interest;- ✓ Expert presenter at the First panle meeting; *Panel members who has to resign their roles due to work commitmnets and requested to support
