# Supplementary material for: Psychosocial recommendations for the care of children and adults with epidermolysis bullosa and their family: evidence based guidelines
Source: Orphanet J Rare Dis. 2019 Jun 11;14:133. doi: 10.1186/s13023-019-1086-5 (PMC6560722; doi:10.1186/s13023-019-1086-5)
Supplement: Supplementary file 2 — Copy of Appraisal and bias Tool. (PDF 638 kb) [file 13023_2019_1086_MOESM2_ESM.pdf]

Psychosocial CPG: Appendix 2

PART ONE: ELIGIBILITY		Answer	
**Participants**	**Do the participants in the study have EB, are family members of someone with EB, are carers (professional/personal) of person(s) with EB? *(what proportion sufficient for inclusion?)***	Yes (1) /No (0)	
**Outcomes**	**Is the outcome(s) one or some of the following: Cope, Pain, Wellbeing, Quality of life, Access to professional, Individual, Family support, independence?**	Yes (1) /No (0)	
**Design**	**Is the methdology one of the following: Quantitative, Qualitative, Systemic reviews, Meta-analysis, RCTs, Cohorts, Case control, Diagnostic studies, Observational? If the paper is a dissertation, position (opinion) paper or a study validating general quality of life measure answer NO (we are excluding these papers)**	Yes (1) /No (0)	
	**ACTION**	If you have answered **NO** to any of these questions please **STOP**	**If you have answered YES** for all questions, please proceed to **Part**

PART TWO: QUALITY ASSESSMENT		Answer	
**Overview**	**1. Does the title reflect the content?**	Yes (1) /No (0)	
	**2. Are the authors contact details and institute reported?**	Yes (1) /No (0)	
	**3. Does the abstract summarise the key components?**	Yes (1) /No (0)	
	**4. Is the rationale for undertaking the research clearly outlined?**	Yes (1) /No (0)	
	**5. Has there been a comprehensive literature review with a clearly outlined process?**	Yes (1) /No (0)	
	**6. Is the aim of the research clearly stated?**	Yes (1) /No (0)	
	**7. Has it been approved by an ethical board?**	Yes (1) /No (0)	
**Sampling (BIAS)** Is the sample representative of the populations of interest i.e., people with EB, family members of people with EB, people who work with people with EB?			
**Was everyone included who should be included? Or was there something particular (biased) about the sample? E.g., only people with RDEB? Or, only people with hand problems? Only people who have been referred to a psychologist?**		Yes (1) /No (0)	
	**What are the sources of sampling bias..........**		
**Method Design Section A**	**Is this Paper Quantitative research? (including mixed method)**	If you have answered NO go to section B.	Yes complete the quantitative section A
	**8. Those this study have a control group?**	Yes (1) /No (0)	
	**9. Is the study design clearly identified, and is the rationale for choice of design evident?**	Yes (1) /No (0)	
	**10. Is there an experimental hypothesis clearly stated?**	Yes (1) /No (0)	
	**11. Are the key variables clearly defined?**	Yes (1) /No (0)	
	**12. Were the outcome measures valid? Did they actually measure what the study set out to measure?**	Yes (1) /No (0)	
**Method Design Section B**	**Is this a paper on Qualitative research? (including mixed method)**	If you have answered NO go to FINAL section.	Yes complete the qualitative section B
	**8. Is the study design clearly identified, and**	Yes (1) /No (0)	
	**9. Is the selection of participants described and the sampling method identified?**	Yes (1) /No (0)	
	**10. Is the method of data analysis credible and confirmable?**	Yes (1) /No (0)	
	**11. Are the results presented in a way that is appropriate and clear?**	Yes (1) /No (0)	
**Final section**			
	**12. Is there a good critical discussion linked to relevant evidence?**	Yes (1) /No (0)	
	**13. Are the results generalizable?**	Yes (1) /No (0)	
	**14. Are the results transferable?**	Yes (1) /No (0)	
	**15. Is the conclusion comprehensive?**	Yes (1) /No (0)	
	**16. Have they reported any limitations to the study?**	Yes (1) /No (0)	
	**17. Have there any conflicts of interest with the authors or the funding?**	Yes (1) /No (0)	
**Final Quality Framework questions (these need discussion in your outcome groups)**			
**Per outcome summary table**	**Sample populations: What type of EB**		
	**Number of subjects with EB**		
	**Study Design/ Method**		
	**18. Was the chosen study design appropriate to answer the question posed in the study?**	Yes (1) /No (0)	
	**19. Is the method of data analysis valid and reliable?**	Yes (1) /No (0)	
	**20. Did the measurements have proven satisfactory reliability and validity?**	Yes (1) /No (0)	
**Framework totals**	**Maximum score for study types**		
	**Qualitative studies**	20	
	**Quantitative studies**	21	
	**Mixed methods studies**	25	
**How much population sample selection bias is present**			
**1. Does the study use random selection to put participants into groups?**		Yes (1) /No (0)	
**2. Is the person undertaking the research or data analysis unknown to the subject e.g. involved in their care?**		Yes (1) /No (0)	Maximum score of 4 for % bias
**3. Are all patients in study reported on**		Yes (1) /No (0)	
**4. Is there Selective out-come reporting? e.g. not all outcomes or patients are reported on**		Yes (1) /No (0)	

Rating the Strength of Psychosocial Outcome article summaries & Recommendations	
**Does this OUTCOME address a subject that is a priority for our patient population**?	**1.** very weak, **2.** somewhat weak, **3.** somewhat strong, **4.** very strong
**How strong is the evidence** that what the article is saying is true = the overall	**1.** very weak, **2.** somewhat weak, **3.** somewhat strong, **4.** very strong
**Look at SIGN** and quality score to do this and consider any **grey literature**.	**1.** very weak, **2.** somewhat weak, **3.** somewhat strong, **4.** very strong
**Are the desired effects relevant**? Discuss	**1.** very weak, **2.** somewhat weak, **3.** somewhat strong, **4.** very strong
**Are there any undesirable effects**? Discuss	**1.** very weak, **2.** somewhat weak, **3.** somewhat strong, **4.** very strong
**Are the costs small relative to the benefits** in carrying out the recommendations?	**1.** very weak, **2.** somewhat weak, **3.** somewhat strong, **4.** very strong
**Are the recommendations feasible to implement**?	**1.** very weak, **2.** somewhat weak, **3.** somewhat strong, **4.** very strong
