# Supplementary material for: Psychosocial recommendations for the care of children and adults with epidermolysis bullosa and their family: evidence based guidelines
Source: Orphanet J Rare Dis. 2019 Jun 11;14:133. doi: 10.1186/s13023-019-1086-5 (PMC6560722; doi:10.1186/s13023-019-1086-5)
Supplement: Supplementary file 3 — CPG Evaluation Form: Pre implementation. (PDF 138 kb) [file 13023_2019_1086_MOESM3_ESM.pdf]

Psychosocial Guidelines: Appendix 3

CPG Evaluation Form: Pre implementation

Please answer the questions below to help us evaluate the usefulness and effectiveness of our guidelines, as well as learn more about those using the information contained in the publication.

<table>
<tr><th colspan="4">Section A: General Information</th></tr>
<tr><td>How did you hear about this guideline?
Please tick all that apply and provide details where applicable</td><td colspan="3">☐ Journal searches (e.g. PubMed)
☐ Event (e.g. DEBRA conference, EB study day)
☐ Recommendation (e.g. family member, clinician)
☐ DEBRA communication (e.g. newsletter, website, social media)
☐ Other (please specify)</td></tr>
<tr><td>Why have you chosen to review this guideline?</td><td colspan="3"></td></tr>
<tr><td>City and country:
Residence</td><td colspan="3"></td></tr>
<tr><td>DEBRA group affiliation:
If applicable</td><td colspan="3"></td></tr>
<tr><td>Which of the following best describes you?
Please tick all that apply</td><td>Group A
Professionals
☐ Clinician
☐ Allied health professional
☐ Professional carer</td><td>Group B
Non-professionals
☐ Person living with EB
☐ *Caregiver (family member)
☐ Caregiver (friend)
☐ Friend</td><td>Group C
Other
☐ Other (please provide details)</td></tr>
<tr><td></td><td>Please continue with Section B</td><td>Please continue with Section C</td><td>Please continue with Section B or C (as appropriate)</td></tr>
</table>

<table>
<tr><th colspan="2">Section B: Professionals</th></tr>
<tr><td>Your institute:
Name and address (including country)</td><td></td></tr>
<tr><td>Tell us about the type of EB services you offer:
Please tick all that apply and provide details</td><td>☐ Hospital ☐ Community ☐ Home service ☐ Other (please specify)</td></tr>
</table>

* For the purposes of this document, a caregiver indicates a non-professional. If you are a carer in a professional capacity, please tick the ‘Professional carer’ option in Group A.

debra
International.

Psychosocial Guidelines: Appendix 3

Please tell us who works with EB patients at your institute:

Please tick all that apply and circle the number of relevant people

☐ Consultant	1	2	3	4+
☐ Dermatologist	1	2	3	4+
☐ Surgeon	1	2	3	4+
☐ Nurse (specialist)	1	2	3	4+
☐ Psychologist, psychotherapist	1	2	3	4+
☐ Dietician, nutritionist	1	2	3	4+
☐ Dentist	1	2	3	4+
☐ Physiotherapist	1	2	3	4+
☐ Podiatrist	1	2	3	4+
☐ Occupational therapist, hand therapist	1	2	3	4+
☐ Social worker/social support	1	2	3	4+
☐ Other (please specify)	1	2	3	4+

Please give a summary of the EB service(s) your institute provides:

300 words maximum; please attach and use additional pages, if needed

Target groups:

Please tick all that apply and provide approximate numbers, where applicable

**Living with EB (years of age)**	**EBS**	**JEB**	**DDEB**	**RDEB**	**KS**
☐ Newborn and neonatal					
☐ Children (0-7)					
☐ Children (8-12)					
☐ Teenagers (12-18)					
☐ Young adults (18-25)					
☐ Adults (25+)					
☐ Other (please specify)					

Before reading the guideline, what information do you/your team already know about this clinical issue?

Please circle the number you feel best reflects your experience and/or tick the most appropriate box, where applicable

Knowledge about this clinical issue:

**1**	**2**	**3**	**4**	**5**
None				Expert

	***Yes**	**No**
Do you have an EB specialist working with you?	☐	☐
*If yes, do they have expertise in the guideline topic?	☐	☐

* If yes, Please provide details:

Psychosocial Guidelines: Appendix 3

<table>
<tr><td></td><td>How confident are you/your team in providing care for EB patients relating to this issue?

1 Not at all confident   2   3   4   5 Extremely confident

How often do you/your team manage this clinical issue?

1 Less than yearly   2 Yearly   3 Monthly   4 Weekly   5 Daily</td></tr>
<tr><td>Describe how you think this guideline will benefit your service users:
Including patients, clients, families, etc.</td><td></td></tr>
</table>

<table>
<tr><th colspan="2">Section C: Non-professionals</th></tr>
<tr><td>Which type of EB do you (your family member/friend) have?</td><td>☐ EBS   ☐ JEB   ☐ DDEB   ☐ RDEB   ☐ KS
☐ I (my family member/friend) have not officially been diagnosed
☐ Other (please specify)</td></tr>
<tr><td>Before reading the guideline, what information do you already know about this clinical issue?
Please circle the number you feel best reflects your experience and/or tick the most appropriate box, where applicable</td><td>How confident are you to get appropriate care in this clinical area?

1 Not at all confident   2   3   4   5 Extremely confident

Why did you indicate this level of confidence?
☐ No one is available to help
☐ I only have written information about the clinical area
☐ I have a person I can reach by phone, email, or other means of communication
☐ I only have a local, general doctor
☐ My local EB association gives me support (please specify)
☐ My doctor has experience
☐ My centre has EB experts
☐ I do not require care
☐ Other (please specify)</td></tr>
</table>

Psychosocial Guidelines: Appendix 3

	How often do you (your family member/friend) experience this problem?<br><br>**1** Less than yearly — **2** Yearly — **3** Monthly — **4** Weekly — **5** Daily<br><br>How do you (your family member/friend) normally manage this problem?

DEBRA International (DI) would like to further contact respondents to this guideline survey a year later for post evaluation measurement. The purpose of this additional survey is to evaluate the use of the guideline after its principles have been applied in practice.

Declaration

I understand that the information I provide will be used for data collection and guideline evaluation purposes only.

I understand that all data collected through these surveys will be anonymised and will not become identifiable in the evaluation process.

I understand that if DI would like to contact me in the future, they will only be able to do so for the purposes of being asked to complete the post evaluation survey, unless consent has otherwise been obtained for additional purposes.

I understand I am under no obligation to complete the post evaluation survey once I receive it, and that I am able to change or withdraw my consent at any time by notifying DI (office@debra-international.org). I understand if consent is withdrawn, DI will destroy all identifiable records (i.e. contact details provided with the pre or post evaluation surveys).

I understand I have the right to ask to see any information and records held about me by DI.

☐ I give my consent for DI to contact me after one year with a post evaluation guideline survey.

For more details on how DI uses your information, please visit www.debra-international.org.

**Contact Details**			
Full name			
Signature		Date	
Email			
